# Synthesis of Bicyclic Isoxazoles and Isoxazolines via Intramolecular Nitrile Oxide Cycloaddition

**DOI:** 10.3390/molecules200610910

**Published:** 2015-06-12

**Authors:** Wen-Chang Chen, Veerababurao Kavala, Yu-Hsuan Shih, Yu-Hsuan Wang, Chun-Wei Kuo, Tang-Hao Yang, Chia-Yu Huang, Hao-Hsiang Chiu, Ching-Fa Yao

**Affiliations:** Department of Chemistry, National Taiwan Normal University, 88, Sec. 4, Ting-Chow Road, Taipei 116, Taiwan; E-Mails: wcchen@taigenbiotech.com (W.-C.C.); kavalaveeru@gmail.com (V.K.); coshih@gmail.com (Y.-H.S.); baby11710@hotmail.com (Y.-H.W.); cwkuo.water@gmail.com (C.-W.K.); a129084303xx@gmail.com (T.-H.Y.); tlotras@gmail.com (C.-Y.H.); a210627@hotmail.com (H.-H.C.)

**Keywords:** bicyclic isoxazole/isoxazolines, cycloaddition, nitrile oxide, nitroalkane, Yamaguchi reagent

## Abstract

An efficient and straight forward procedure for the syntheses of bicyclic isoxazole/isoxazoline derivatives from the corresponding dimethyl-2-(2-nitro-1-aryl/alkyl)-2-(prop-2-yn-1yl)malonates or dimethyl 2-allyl-2-(2-nitro-1-aryl/alkyl ethyl)malonate is described. High yields and simple operations are important features of this methodology.

## 1. Introduction

Isoxazole and isoxazoline derivatives are important scaffolds found in many naturally occurring and biologically active compounds [1,2,3,4,5]. They are considered to be important precursors for the synthesis of β-hydroxyketones or β-aminoalcohols, α,β-unsaturated ketones, and many other valuable compounds [6,7,8,9,10]. Isoxazole or isoxazoline motifs fused with carbocycles or heterocycles are known to possess a variety of bioactivities and are also useful surrogates for the generation of various other bioactive compounds [11,12,13,14,15].

Isoxazole and isoxazoline derivatives are typically synthesized through *in situ* formation of a nitrile oxide followed by an intramolecular dipolar cycloaddition [16,17,18,19]. The most common methods for generating nitrile oxides are the dehydrohalogenation of hydroximoyl chlorides [20,21,22,23,24], the oxidation of aldoximes [25,26,27,28,29,30,31,32], and the dehydration of nitroalkanes [33,34,35,36]. Although a wide variety of procedures are available for the synthesis of diverse isoxazole and isoxazoline derivatives, few of them address the preparation of carbocyclic fused isoxazole/isoxazoline derivatives [37,38,39,40,41,42]. The dehydration of the nitroalkanes followed by intramolecular dipolar cycloaddition is the most popular method for the construction of carbocycle fused isoxazole/isoxazoline derivatives. The literature procedures for the generation of carbocycle fused isoxazole/isoxazoline from various nitroalkane derivatives [43,44,45,46,47] involve the use of phenyl isocyanate and triethylamine, treatment of the nitronate with di-*tert*-butyl dicarbonate in the presence of catalytic amounts of dimethylaminopyridine (DMAP), the use of chlorformates in the presence trimethylamine and trimethylsilyl chloride (TMSCl) in the presence of trimethylamine. We also reported on a different procedure for the construction of bicyclicisoxazole/isoxazoline derivatives by using trichlorotriazine (TCT), chloroformate and phenyl isocyante as dehydrating agents. Although a few methods for the synthesis of carbocycle fused isoxazole/isoxazoline derivatives are efficient, some of the available methods suffer from drawbacks such as low yields and selectivity and long reaction times. Hence, a simple and straightforward method for generating such derivatives in high yields and in better selectivity would be highly desirable. It is well known that the Yamaguchi reagent (2,4,6-trichlorobenzoyl chloride) is a dehydrating agent used in the construction of macrolides and highly functionalized esters [48,49,50]. However, to our knowledge this reagent has not been utilized for the dehydration of nitroalkanes. Herein, we wish to report on the synthesis of bicyclicisoxazole/isoxazoline derivatives from the corresponding dimethyl 2-propargyl-2-(2-nitro-1-aryl/alkylethyl)malonate/dimethyl 2-allyl-2-(2-nitro-1-aryl/alkyl ethyl)malonates using the Yamaguchi reagent as a dehydrating agent.

## 2. Results and Discussion

We first synthesized the starting materials, including the dimethyl 2-propargyl-2-(2-nitro-1-aryl/alkylethyl)malonates **1a**–**15a** or dimethyl 2-allyl-2-(2-nitro-1-aryl/alkyl ethyl)malonates **1c**–**6c** from the corresponding nitroalkenes and propargylmalonate/allylmalonate derivatives in the presence of potassium tertiary butoxide (*t*-BuOK) and lithium bis(trimethylsilyl)amide (LHMDS) [34,43]. We then initiated our studies of the reaction by using dimethyl-2-(2-nitro-1-phenylethyl)-2-(prop-2-yn-1yl)malonate (**1a**) as the substrate.

To start the reaction, **1a** was treated with potassium *tert*-butoxide (2.5 equiv.) and the Yamaguchi reagent (2,4,6-trichlorobenzoyl chloride) in dichloromethane at −78 °C. The corresponding bicyclic isoxazole product was produced in 65% yield after 24 h. Encouraged by this initial result, we next focused our attention on optimizing the reaction conditions for the formation the bicyclic isoxazole product. To find a superior base for this transformation the reaction, various bases, including trimethylamine (Et_3_N), 1,8-diazabicycloundec-7-ene (DBU), and dimethylaminopyridine (DMAP) were screened. Among them, DBU was found to be the most suitable one. As the Yamaguchi reagent functions as a dehydrating agent in this reaction, we were curious to examine whether other benzoyl chlorides could be used for this purpose. To check this, we reacted **1a** with various benzoyl chlorides including unsubstituted, 2-chloro-, and 3,4-dichlorobenzoyl chlorides in the presence of DBU. All of these reactions resulted in the production of the desired product in moderate to good yields. However, the yield of the desired product was higher when the Yamaguchi reagent was used as the dehydrating agent. Although the yield of the product was satisfactory, the reaction time was longer when a base (2.5 equiv.) and a dehydrating agent (3 equiv.) was used.

On the other hand, we observed that the efficiency of the nitroalkane to nitrile oxide dehydration reaction was enhanced in the presence a catalytic amount of Lewis acid [43] and conducting the reaction in the presence of a Lewis acid produced the corresponding product in a very short time. Hence, we tested various Lewis acid catalysts, including SnCl_4_, ZnCl_2_ and ZrCl_4_. Among the Lewis acids tested, ZrCl_4_ was found to the best choice. Further, to determine the optimum amount of base required for this reaction, we carried out the reaction using 2 equiv., 1.5 equiv., and 1.0 equiv. of DBU. The reaction was the most efficient when more than 1.5 equivalent of base was used. Moreover, the reaction resulted in excellent yields and the reaction time was shorter when a base (1.5 equiv.) and 1.5 equiv. of the Yamaguchi reagent was used. To determine the effect of temperature, we conducted the reactions at 0 °C and at room temperature. The reaction furnished the desired bicyclic isoxazole derivative in moderate yield when it was conducted at 0 °C, whereas a poor yield of the bicyclic isoxazole was obtained when the reaction was carried out at room temperature. Thus, the optimum reaction conditions for the formation of the bicyclic isoxazole were determined to be the use of 1.5 equiv. DBU, 1.5 equiv. of the Yamaguchi reagent and 10 mol % ZrCl_4_ as the catalyst in DCM at −78 °C. These optimization studies are summarized in Table 1.

**Table 1:** Optimization studies.
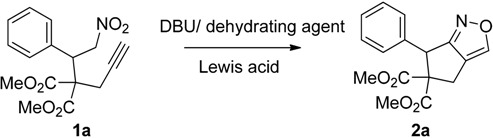

Entry	Base (equiv.)	Dehydrating Agent (equiv.)	Lewis Acid	Temp. (°C)	Time (h)	Yield ^a, b^
1	K *t*-BuO	(2.5)	2,4,6-Trichlorobenzoyl chloride	(3)	—	‒78	24	65
2	TEA	(2.5)	2,4,6-Trichlorobenzoyl chloride	(3)	—	‒78	24	50
3	DMAP	(2.5)	2,4,6-Trichlorobenzoyl chloride	(3)	—	‒78	24	67
4	DBU	(2.5)	2,4,6-Trichlorobenzoyl chloride	(3)	—	‒78	24	73
5	DBU	(2.5)	Benzoyl chloride	(3)	—	‒78	48	62
6	DBU	(2.5)	2-Chlorobenzoyl; chloride	(3)	—	‒78	35	65
7	DBU	(2.5)	3,4-Dichlorobenzoyl chloride	(3)	—	‒78	24	65
8	DBU	(2.5)	2,4,6-Trichlorobenzoyl chloride	(1.5)	ZnCl_2_	‒78	3	82
9	DBU	(2.5)	2,4,6-Trichlorobenzoyl chloride	(1.5)	ZnCl_4_	‒78	4	87
10	DBU	(2.5)	2,4,6-Trichlorobenzoyl chloride	(1.5)	SnCl_4_	‒78	5	60
11	DBU	(2.0)	2,4,6-Trichlorobenzoyl chloride	(1.5)	ZnCl_4_	‒78	4	90
12	DBU	(1.5)	2,4,6-Trichlorobenzoyl chloride	(1.5)	ZnCl_4_	‒78	4	95
13	DBU	(1.0)	2,4,6-Trichlorobenzoyl chloride	(1.5)	ZnCl_4_	‒78	7	85
14	DBU	(1.5)	2,4,6-Trichlorobenzoyl chloride	(1.5)	ZnCl_4_	0	2	40
15	DBU	(1.5)	2,4,6-Trichlorobenzoyl chloride	(1.5)	ZnCl_4_	25	2	20

^a^ NMR yields. ^b^ Reactions carried out in 0.5 mmol scale.

After determining the optimum conditions for the formation of bicyclic isoxazoles, we then utilized these conditions for investigating the scope and limitations of the methodology with various dimethyl-2-(2-nitro-1-aryl/alkyl)-2-(prop-2-yn-1yl)malonates derived from the corresponding nitroalkenes and dimethylproparylmalonate (Table 2).

**Table 2:** Synthesis of bicyclic isoxazole derivatives.
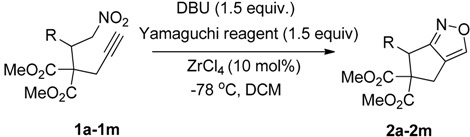

Entry	Substrate	Product	Time (h)	Yield% ^a,b^
1	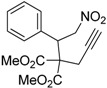 **1a**	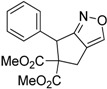 **2a**	4	95
2	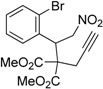 **1b**	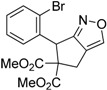 **2b**	2	86
3	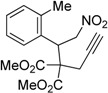 **1c**	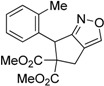 **2c**	3	89
4	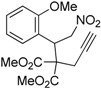 **1d**	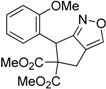 **2d**	3	92
5	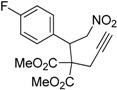 **1e**	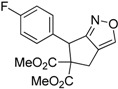 **2e**	5	90
6	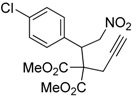 **1f**	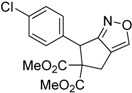 **2f**	5	87
7	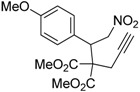 **1g**	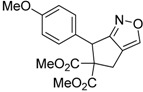 **2g**	3	84
8	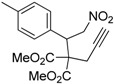 **1h**	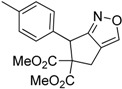 **2h**	3	86
9	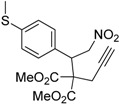 **1i**	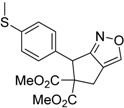 **2i**	3	84
10	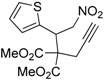 **1j**	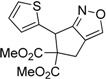 **2j**	15	79
11	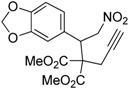 **1k**	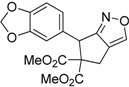 **2k**	7	92
12	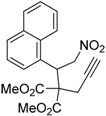 **1l**	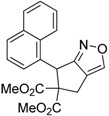 **2l**	12	80
13	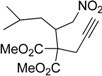 **1m**	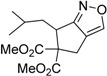 **2m**	5	87

^a^ Isolated yields. ^b^ Reactions performed in 0.5 mmol scale.

Under the optimized conditions, the substrate containing an unsubstituted phenyl group (compound **1a**) gave the corresponding bicyclic isoxazole derivative in excellent yield. The reactions of substrates possessing an electron withdrawing group such as a bromo group (compound **1b**) and substrates containing an electron donating group such as methyl (compound **1c**) and methoxy (compound **1d**) groups at the *ortho* position of the phenyl group reacted smoothly to yield the corresponding bicyclic isoxazole derivatives in excellent yields. On the other hand, substrates containing an electron withdrawing group at the *para* position of the phenyl group such as **1e** and **1f**, provided the corresponding bicycloisoxazole derivatives **2e** and **2f**, respectively, in excellent yields. In addition, substrates possessing electron donating groups (OMe and Me) and an electron neutral group (SMe) at the *para* position of the phenyl group reacted with equal ease to provide the desired products **2g**, **2h** and **2i** in good yields. Further, substrates containing thiophene (compound **1j**), and methyleneoxy groups (compound **1k**) reacted smoothly and provided the corresponding bicyclicisoxazole derivatives **2j** and **2k**, respectively, in good to excellent yields. Substrates containing a naphthalene moiety (compound **2l**) and an alkyl group (compound **2m**) also produced the corresponding products in high yields under the present reaction conditions.

**Scheme 1:**
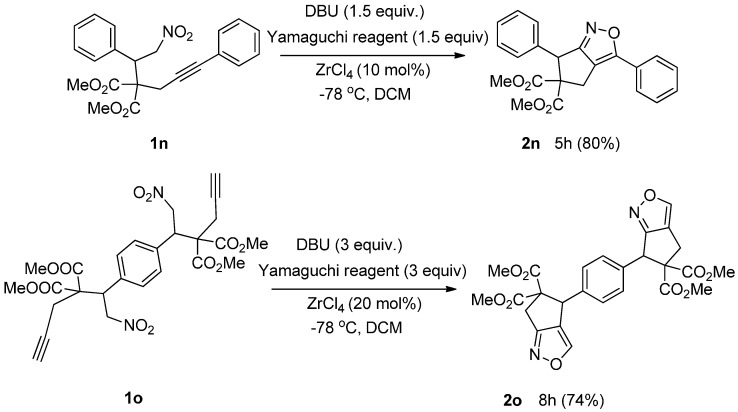
Synthesis of bicyclic isoxazole derivatives.

To extend the scope of this reaction, we examined the reactions of dimethyl 2-(2-nitro-1-phenylethyl)-2-(3-phenylprop-2-yn-1-yl)malonate (**1n**) and tetramethyl 2,2′-(1,4-phenylenebis(2-nitroethane-1,1-diyl))bis(2-(prop-2-yn-1-yl)malonate) (**1o**). To our delight, both reactions provided the corresponding bicyclic isoxazole derivatives (**2n** and **2o**) in good yields (Scheme 1).

We also investigated the reactions of 2-allyl-2-(2-nitro-1-aryl/alkyl ethyl)malonate derivatives under the present reaction conditions and the results are listed in Table 3. As shown in Table 3, the reactions of substrates containing both electron withdrawing as well as electron donating groups exhibited a similar reactivity under the reaction conditions. However, when substrates containing thienyl and naphthyl moieties were used a slightly longer reaction time was needed to produce good yield of the corresponding bicyclic isoxazoline derivatives (Table 3).

It is important to note that the reactions of all 2-allyl-2-(2-nitro-1-aryl/alkylethyl)malonate derivatives resulted in the production of mixture of diastereomers and among the diastereomers, the *cis* isomer was the major component and the *trans* isomer was the minor one. The NMR signal for the methine proton at the bridgehead carbon in the *trans* isomer appears at 4.62 ppm, compared to 3.82 ppm for the *cis* isomer, which permits *trans* and *cis* isomers to be identified. The diastereoslectivity (*cis*:*trans*) ranged from 5.6:1 to 3.6:1. The diasteroslectivity for this reaction slightly better than in our previous report [43]. These results were consistent with the our previous results as well as other literature reports [43,51,52].

**Table 3:** Synthesis of bicyclic isoxazoline derivatives.
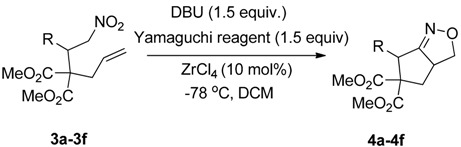

Entry	Substrate	Product	Time (h)	Yield% ^a,b^	*cis:trans ^c^*
1	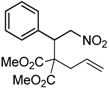 **3a**	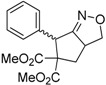 **4a**	3	88	5.3:1
2	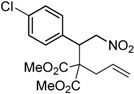 **3b**	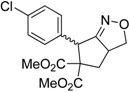 **4b**	4	83	4.6:1
3	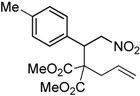 **3c**	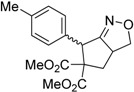 **4c**	5	81	3.6:1
4	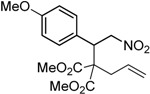 **3d**	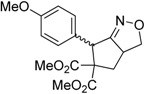 **4d**	5	85	5.4:1
5	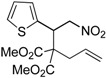 **3e**	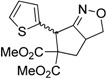 **4e**	12	78	4.8:1
6	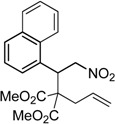 **3f**	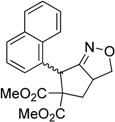 **4f**	7	84	5.6:1

^a^ Isolated yields. ^b^ Reactions performed in 0.5 mmol scale. ^c^ *cis*:*trans* isomers were determined from ^1^H-NMR.

**Scheme 2:**
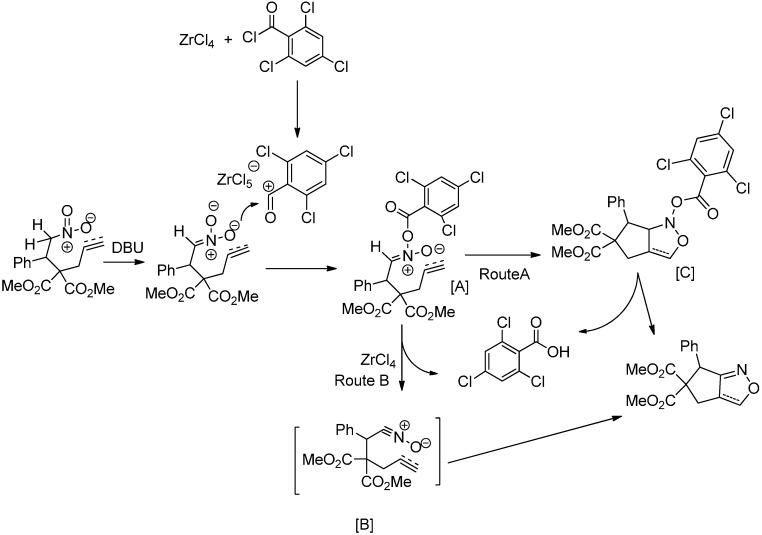
Plausible mechanistic pathways.

Based on our experience with nitroalkanes and the literature reports related to the Yamaguchi reagent, the formation of bicyclic isoxazole/isoxazolines from dimethyl 2-propargyl-2-(2-nitro-1-aryl/alkylethyl)malonates or dimethyl 2-allyl-2-(2-nitro-1-aryl/alkylethyl)malonates with a base and the Yamaguchi reagent in the presence of an acidic catalyst could be explained by two mechanistic pathways shown in Scheme 2. The reaction of the nitroalkane in the presence a base could result in the production of a nitronate ion, which could be further converted into a benzoylnitronate [A] by reaction with the Yamaguchi reagent and zirconium chloride. This benzoylnitronate [A] could then be converted into the corresponding nitrile oxide [B] intermediate in the presence of zirconium chloride, which would further undergo intramolecular nitrile oxide cycloaddition (INOC) to afford the desired carbocycle fused isoxazole/isoxazoline (Scheme 2, Route B). Another possibility is that the benzoylnitronate might act as a 1,3-dipole [53] to undergo INOC to obtain intermediate [C], which upon losing trichlorobenzoic acid, would produce the desired bicyclic compound (Scheme 2, Route A). 

## 3. Experimental Section 

### 3.1. General Information

All chemicals were purchased from various commercial sources and used directly without further purification. Analytical thin-layer chromatography was performed using E. Merck (New York, NY, USA) silica gel 60F glass plates and E. Merck silica gel 60 (230–400 mesh) was used in flash chromatography separations. MS were measured by a JMS-HX110 spectrometer (JEOL, Hsinchu, Japan). HRMS spectra were recorded using ESI-TOF or EI^+^ mode or FAB^+^. ^1^H- (400 MHz) and ^13^C-NMR (100 MHz) spectra were recorded with an Advance EX 400 MHz spectrometer (Bruker, San Francisco, CA, USA). Chemical shifts are reported in parts per million (δ) using TMS as an internal standard and coupling constant were expressed in hertz. IR spectra were performed on a 100 series FT-IR instrument (Perkin Elmer, Waltham, MA, USA). Melting points were recorded using an capillary melting point apparatus (Electrothermal, Staffordshire, UK) and are uncorrected. All substrates were prepared using literature procedures.

### 3.2. General Procedure for the Preparation of **1a**–**1o**

Potassium *tert*-butoxide (3 mmol) was added to a solution of dimethyl propargylmalonate (2.4 mmol) in anhydrous THF (15 mL) at room temperature under nitrogen atmosphere. The reaction mixture was then cooled to −78 °C and a solution of the nitroalkene (2 mmol) in anhydrous THF (5 mL) was added dropwise over a period of five minutes. The reaction mixture was then stirred at −78 °C until the reaction was complete, which was monitored by TLC. A saturated aqueous NH_4_Cl was then added and the resulting mixture extracted with ethyl acetate (3 × 10 mL). The combined organic phases were dried over MgSO_4_ and the solvent removed *in vacuo*. The residue was purified by flash column chromatography over silica (ethyl acetate and hexane).

*Dimethyl 2-(1-(2-bromophenyl)-2-nitroethyl)-2-(prop-2-yn-1-yl)malonate* (**1b**): Colorless solid; Yield: 67% (5 h); m.p. 100–101 °C; ^1^H-NMR (CDCl_3_) δ 7.60 (d, *J* = 8.0 Hz, 1H), 7.30 (t, *J* = 7.6 Hz, 1H), 7.20–7.14 (m, 2H), 5.22 (d, *J* = 8.0 Hz, 1H), 5.16–5.03 (m, 2H), 3.82 (s, 3H), 3.80 (s, 3H), 2.76 (m, 2H), 2.15 (s, 1H); ^13^C-NMR (CDCl_3_) δ 169.2, 169.1, 134.8, 134.2, 130.1, 128.4, 128.2, 127.3, 78.8, 78.0, 72.8, 60.8, 53.6, 53.3, 44.8, 23.8; HRMS (EI) *m*/*z* calc. for C_16_H_16_NO_6_^79^Br (M^+^) 397.0161, found 397.0156.

*Dimethyl 2-(2-nitro-1-(o-tolyl)ethyl)-2-(prop-2-yn-1-yl)malonate* (**1c**): Colorless solid; Yield: 75% (4 h); m.p. 98–99 °C. ^1^H-NMR (CDCl_3_) δ 7.17–7.12 (m, 3H), 6.92–6.90 (m, 1H), 5.32 (dd, *J* = 6.7, 3.1 Hz, 1H), 4.96 (dd, *J* = 11.6 Hz, 6.6 Hz, 1H), 4.81 (dd, *J* = 5.6 Hz,, 2.9 Hz, 1H), 3.83 (s, 3H), 3.78 (s, 3H), 2.85 (dd, *J* = 8.7 Hz, 2.8 Hz, 1H), 2.51 (s, 3H), 2.41 (dd, *J* = 8.7 Hz, 2.6 Hz, 1H), 2.15 (t, *J* = 2.7 Hz, 1H); ^13^C-NMR (CDCl_3_) δ 169.5, 169.4, 139.2, 133.7, 131.7, 128.5, 126.6, 126.4, 79.2, 79.0, 73.2, 60.8, 53.4, 53.3, 40.8, 23.7, 20.1; HRMS (EI) *m*/*z* calc. for C_17_H_19_NO_6_ (M^+^) 372.1059, found 372.1069.

*Dimethyl 2-(2-nitro-1-(2-methoxyphenyl)ethyl)-2-(prop-2-yn-1-yl)malonate* (**1d**): Colorless solid; Yield: 72% (4 h); m.p. 141–142 °C. ^1^H-NMR (CDCl_3_) δ 7.26–7.22 (m, 2H), 6.88–6.81 (m, 2H), 5.17 (d, *J* = 5.8 Hz, 2H), 4.65 (s, 1H), 3.77 (s, 3H), 3.75 (s, 3H), 3.71 (s, 3H), 2.80 (d, *J* = 17.1 Hz, 1H), 2.30 (d, *J* = 16.3 Hz, 1H), 2.11 (s, 1H); ^13^C-NMR (CDCl_3_) δ 169.5, 169.1, 157.6, 133.0, 130.0, 122.3, 120.9, 111.2, 78.6, 76.4, 72.6, 59.0, 55.3, 53.2, 52.7, 44.0, 24.3.

*Dimethyl 2-(1-(4-fluorophenyl)-2-nitroethyl)-2-(prop-2-yn-1-yl)malonate* (**1e**): Colorless solid; Yield: 84% (3 h); m.p. 89–90 °C. ^1^H-NMR (CDCl_3_) δ 7.18 (dd, *J* = 8.4, 5.3 Hz, 2H), 7.00 (t, *J* = 8.5 Hz, 2H), 5.28 (dd, *J* = 13.6, 3.0 Hz, 1H), 4.96 (dd, *J* = 13.5, 11.6 Hz, 1H), 4.50 (dd, *J* = 11.7, 2.9 Hz, 1H), 3.80 (s, 3H), 3.78 (s, 3H), 2.79 (dd, *J* =17.5 Hz, 2.5 Hz, 1H), 2.37 (dd, *J* =17.5 Hz, 2.5 Hz, 1H), 2.23 (t, *J* = 2.3 Hz, 1H); ^13^C-NMR (CDCl_3_) δ 169.1, 169.0, 164.0 (d, *J_C-F_* = 245.0), 130.7 (d, *J_C-F_* = 8.0), 130.6 (d, *J_C-F_* = 4.0), 116.3 (d, *J_C-F_* = 21.0), 78.0, 76.9, 73.8, 59.7, 53.6, 53.4, 44.9, 24.1; HRMS (EI) *m*/*z* calc. for C_16_H_16_NO_6_F (M^+^) 337.0963, found 337.0969.

*Dimethyl 2-(1-(4-chlorophenyl)-2-nitroethyl)-2-(prop-2-yn-1-yl)malonate* (**1f**): Colorless solid; Yield: 98% (5 h); m.p. 123–124 °C; ^1^H-NMR (CDCl_3_) δ 7.30 (d, *J* = 8.3 Hz, 2H), 7.15 (d, *J* = 8.3 Hz, 2H), 5.30 (dd, *J* = 13.8, 2.9 Hz, 1H), 4.97 (dd, *J* = 13.8, 11.7 Hz, 1H), 4.51 (dd, *J* = 11.7, 2.9 Hz, 1H), 3.81 (s, 3H), 3.79 (s, 3H), 2.81 (dd, *J* =17.5 Hz, 2.2 Hz, 1H), 2.37 (dd, *J* =17.5 Hz, 2.2 Hz, 1H), 2.25 (s, 1H); ^13^C-NMR (CDCl_3_) δ 168.9, 168.8, 134.8, 133.2, 130.2, 129.3, 77.9, 77.5, 73.9, 59.5, 53.6, 53.4, 44.9, 24.1; HRMS (EI) *m*/*z* calc. for C_16_H_16_NO_6_^35^Cl (M^+^) 353.0666, found 353.0661.

*Dimethyl 2-(1-(4-methoxyphenyl)-2-nitroethyl)-2-(prop-2-yn-1-yl)malonate* (**1g**): Colorless solid; Yield: 81% (2 h); m.p. 111–112 °C; ^1^H-NMR (CDCl_3_) δ 7.11 (d, *J* = 8.5 Hz, 2H), 6.84 (d, *J* = 8.5 Hz, 2H), 5.27 (dd, *J* = 13.5, 2.9 Hz, 1H), 4.98 (dd, *J* = 13.5, 11.4 Hz, 1H), 4.46 (dd, *J* = 11.4, 2.9 Hz, 1H), 3.81 (s, 3H), 3.79 (s, 3H), 3.77 (s, 3H), 2.79 (dd, *J* =17.4 Hz, 2.2 Hz, 1H), 2.42 (dd, *J* =17.4 Hz, 2.2 Hz, 1H), 2.22 (s, 1H); ^13^C-NMR (CDCl_3_) δ 169.2, 169.1, 159.9, 130.0, 126.4, 114.5, 78.3, 77.9, 73.5, 59.9, 55.4, 53.4, 53.3, 45.0, 24.1; HRMS (EI) *m*/*z* calc. for C_17_H_19_NO_7_ (M^+^) 349.1162, found 349.1156.

*Dimethyl 2-(2-nitro-1-(p-tolyl)ethyl)-2-(prop-2-yn-1-yl)malonate* (**1h**): Colorless solid; Yield: 92% (2 h); m.p. 91–92 °C; ^1^H-NMR (CDCl_3_) δ 7.15 (d, *J* = 8.0 Hz, 2H), 7.05 (d, *J* = 8.0 Hz, 2H), 5.28 (dd, *J* = 13.6, 3.0 Hz, 1H), 5.00 (dd, *J* = 13.6, 11.6 Hz, 1H), 4.47 (dd, *J* = 11.6, 2.9 Hz, 1H), 3.81 (s, 3H), 3.78 (s, 3H), 2.78 (dd, *J* =17.4 Hz, 2.4 Hz, 1H), 2.41 (dd, *J* =17.4 Hz, 2.4 Hz, 1H), 2.30 (s, 3H), 2.23 (s, 1H); ^13^C-NMR (CDCl_3_) δ 169.2, 169.1, 138.7, 131.5, 129.8, 128.6, 78.3, 77.8, 73.5, 59.7, 53.4, 53.3, 45.2, 24.1, 21.2; HRMS (EI) *m*/*z* calc. for C_17_H_19_NO_6_ (M^+^) 333.1212, found 333.1207.

*Dimethyl 2-(2-nitro-1-(4-methylthiophenyl)ethyl)-2-(prop-2-yn-1-yl)malonate* (**1i**): Yellow oil; Yield: 88% (2 h); ^1^H-NMR (CDCl_3_) δ 7.16 (d, *J* = 8.4 Hz, 2H), 7.09 (d, *J* = 8.4 Hz, 2H), 5.26 (dd, *J* = 13.7, 3.1 Hz, 1H), 4.97 (dd, *J* = 13.7, 11.4 Hz, 1H), 4.46 (dd, *J* = 11.4, 3.1 Hz, 1H), 3.79 (s, 3H), 3.77 (s, 3H), 2.78 (dd, *J* =17.4 Hz, 2.6 Hz, 1H), 2.43 (s, 3H), 2.39 (dd, *J* =17.4, 2.4 Hz, 1H), 2.22 (t, *J* =2.6 Hz, 1H); ^13^C-NMR (CDCl_3_) δ 169.1, 169.0, 139.6, 131.1, 129.2, 126.6, 78.2, 77.7, 73.6, 59.7, 53.4, 53.3, 45.1, 24.1, 15.4; HRMS (EI) *m*/*z* calc. for C_17_H_19_NO_6_S (M^+^) 365.0933, found 365.0928.

*Dimethyl 2-(2-nitro-1-(thiophen-2-yl)ethyl)-2-(prop-2-yn-1-yl)malonate* (**1j**): Colorless solid; Yield: 90% (2.5 h); m.p. 101–102 °C; ^1^H-NMR (CDCl_3_) δ 7.25 (d, *J* = 5.0 Hz, 1H), 7.00 (d, *J* = 3.6 Hz, 1H), 6.95 (dd, *J* = 5.0, 3.6 Hz, 1H), 5.26 (dd, *J* = 13.4, 2.6 Hz, 1H), 4.97 (dd, *J* = 13.4, 11.1 Hz, 1H), .4.86 (d, *J* = 11.1 Hz, 1H), 3.83 (s, 3H), 3.79 (s, 3H), 2.89 (dd, *J* = 17.5, 2.7 Hz, 1H), 2.61 (dd, *J* = 17.4, 2.7 Hz, 1H); 2.23 (t, *J* = 2.7 Hz, 1H); ^13^C-NMR (CDCl_3_) δ 168.8, 168.7, 136.8, 128.6, 127.2, 126.3, 79.1, 78.0, 73.6, 60.1, 53.6, 53.5, 41.8, 24.1; HRMS (EI) *m*/*z* calc. for C_14_H_15_NO_6_S (M^+^) 325.0620, found 325.0615.

*Dimethyl 2-(1-benzo[d]*[1,3]*dioxol-5yl)-2-nitroethyl)-2-(prop-2-yn-1-yl)malonate* (**1k**): Colorless solid; Yield: 95% (4 h); m.p. 169–170 °C. ^1^H-NMR (CDCl_3_) δ 6.73 (d, *J* = 8.4 Hz, 1H), 6.67–6.65 (m, 2H), 5.95 (s, 2H), 5.24 (dd, *J* = 13.6, 3.1 Hz, 1H), 4.94 (dd, *J* = 13.6, 11.4 Hz, 1H), 4.42 (dd, *J* = 11.1, 3.1Hz, 1H), 3.81 (s, 3H), 3.78 (s, 3H), 2.81 (dd, *J* = 17.4, 2.6 Hz, 1H), 2.46 (dd, *J* = 17.4, 2.6 Hz, 1H); 2.17 (t, *J* = 2.6 Hz, 1H); ^13^C-NMR (CDCl_3_) δ 169.1, 169.0, 148.2, 148.1, 128.1, 122.6, 109.0, 108.8, 101.5, 78.2, 77.9, 73.6, 59.9, 53.5, 53.4, 45.4, 25.2; HRMS (EI) *m*/*z* calc. for C_17_H_17_NO_8_ (M^+^) 363.0954, found 363.0949.

*Dimethyl 2-(1-(naphthalen-1-yl)-2-nitroethyl)-2-(prop-2-yn-1-yl)malonate* (**1l**): Colorless solid; Yield: 77% (2 h); m.p. 145–146 °C; ^1^H-NMR (CDCl_3_) δ 8.45 (d, *J* = 8.6 Hz, 1H), 7.80 (t, *J* = 9.4 Hz, 2H), 7.54 (t, *J* = 7.6 Hz, 1H), 7.48 (t, *J* = 7.7 Hz, 1H), 7.40 (t, *J* = 7.6 Hz, 1H), 7.24 (d, *J* = 7.2 Hz, 1H), 5.56–5.48 (m, 2H), 5.22 (dd, *J* = 13.5, 10.9 Hz, 1H), 3.80 (s, 3H), 3.63 (s, 3H), 2.78 (dd, *J* = 17.5, 2.6 Hz, 1H), 2.36 (dd, *J* = 17.5, 2.6 Hz, 1H); 2.21 (t, *J* = 2.6 Hz, 1H); ^13^C-NMR (CDCl_3_) δ 169.4, 169.2, 134.1, 132.8, 131.9, 129.5, 128.8, 126.7, 126.3, 125.0, 124.8, 123.8, 79.1, 78.7, 73.3, 60.9, 53.4, 53.3, 39.2, 23.6; HRMS (EI) *m*/*z* calc. for C_20_H_19_NO_6_ (M^+^) 369.1212, found 369.1207.

*Dimethyl 2-(4-methyl-1-nitropentan-2yl-)-2-(prop-2-yn-1-yl)malonate* (**1m**): Pale yellow oil; Yield: 82% (3 h); ^1^H-NMR (CDCl_3_) δ 4.98 (dd, *J* = 14.5, 3.9 Hz, 1H), .4.30 (dd, *J* = 14.5, 5.7 Hz, 1H), 3.75 (s, 3H), 3.74 (s, 3H), 3.36–3.31 (m, 1H), 2.93 (dd, *J* = 17.6, 2.7 Hz, 1H), 2.84 (dd, *J* = 17.6, 2.6 Hz, 1H); 2.11 (t, *J* = 2.7 Hz, 1H), 1.58–1.51 (m, 1H), 1.31–1.18 (m, 2H), 0.93 (d, *J* = 6.4 Hz, 3H), 0.90 (d, *J* = 6.4 Hz, 3H); ^13^C-NMR (CDCl_3_) δ 169.7, 169.4, 78.5, 78.3, 72.9, 60.0, 53.2, 53.1, 39.7, 37.9, 25.7, 23.9, 23.3, 21.4;. HRMS (EI) *m*/*z* calc. for C_14_H_21_NO_6_ (M^+^) 300.1448, found 300.1444.

*Dimethyl 2-(2-nitro-1-phenylethyl)-2-(3-phenylprop-2-yn-1-yl)malonate* (**1n**): Pale yellow oil; Yield: 98% (3 h); ^1^H-NMR (CDCl_3_) δ 7.46–7.45 (m, 2H), 7.35–7.31 (m, 6H), 7.24–7.23 (m, 2H), 5.35 (dd, *J* = 13.7, 3.0 Hz, 1H), 5.06 (dd, *J* = 13.7, 11.3 Hz, 1H), 4.61 (dd, , *J* = 11.2, 3.0 Hz, 1H) 3.84 (s, 3H), 3.80 (s, 3H), 3.02 (d, *J* = 17.5 Hz, 1H), 2.63 (d, *J* = 17.5 Hz, 1H); ^13^C-NMR (CDCl_3_) δ 169.2, 169.1, 134.8, 131.7, 129.0, 128.8, 128.7, 128.5, 128.4, 122.8, 85.5, 83.5, 77.0, 60.1, 53.3, 53.2, 45.7, 24.9;. HRMS (EI) *m*/*z* calc. for C_22_H_21_NO_6_ (M^+^) 395.1369, found 395.1366.

*Tetramethyl 2,2'-(1,4-phenylenebis(2-nitroethane-1,1-diyl))bis(2-(prop-2-yn-1-yl)malonate)* (**1o**): Colorless solid; Yield: 60% (2 h); m.p. 180–182 °C; ^1^H-NMR (CDCl_3_) δ 7.18 (s, 4H), 5.29 (dd, *J* = 13.9, 3.0 Hz, 2H), 5.00 (ddd, *J* = 13.9, 11.1, 3.1 Hz, 2H), 4.48 (d, *J* = 9.9 Hz, 2H), 3.76 (s, 6H), 3.75 (s, 6H), 2.78 (dd, *J* = 17.4, 2.4 Hz, 2H), 2.34 (dt, *J* = 17.4, 3.5 Hz, 2H); 2.20 (s, 2H); ^13^C-NMR (CDCl_3_) δ 169.2, 169.1, 169.0, 135.7, 135.6, 129.5, 78.2, 78.1, 73.8, 73.7, 59.9, 53.5, 53.4, 45.4, 24.2, 24.1; HRMS (ESI) *m*/*z* calc. for C_26_H_28_N_2_O_12_ (M) ^+^ 560.1642, found 560.1642.

### 3.3. General Procedure for the Synthesis of **3a**–**3f**

LHMDS (1.5 mmol) was added to a solution of dimethyl propargylmalonate (1.2 mmol) in anhydrous THF (5 mL) at 0 °C under a nitrogen atmosphere, followed by stirring for 1 h. A solution of nitroalkene (1 mmol) in anhydrous THF (5 mL) was then added drop wise over a period of five minutes at 0 °C. The reaction mixture was then stirred at the same temperature until the reaction was complete, which was monitored by TLC. After the completion of the reaction, a saturated aqueous NH_4_Cl was added and the resulting mixture extracted with ethyl acetate (3 × 10 mL). The combined organic phases were dried over MgSO_4_ and the solvent removed *in vacuo*. The residue was purified by flash column chromatography over silica (ethyl acetate and hexane).

*Dimethyl 2-allyl-2-(1-(4-chlorophenyl)-2-nitroethyl)malonate* (**3b**): Colorless solid; Yield: 98% (2.5 h); m.p. 98–99 °C; ^1^H-NMR (CDCl_3_) δ 7.29 (d, *J* = 8.4 Hz, 2H), 7.07 (d, *J* = 8.4 Hz, 2H), 5.75–5.65 (m, 1H), 5.15–4.91 (m, 4H), 4.18 (dd, *J* = 11.1, 3.1 Hz, 1H), 3.80 (s, 3H), 3.74 (s, 3H), 2.58 (dd, *J* =14.5 Hz, 6.4 Hz, 1H), 2.27 (dd, *J* =14.5 Hz, 8.1 Hz, 1H); ^13^C-NMR (CDCl_3_) δ 170.0, 169.9, 134.7, 133.6, 131.7, 130.4, 129.2, 120.2, 78.2, 60.7, 53.0, 52.9, 46.3, 38.5; HRMS (EI) *m*/*z* calc. for C_16_H_18_NO_6_^35^Cl (M^+^) 355.0823, found 355.0817.

*Dimethyl 2-allyl-2-(2-nitro-1-(p-tolyl)ethyl)malonate* (**3c**): Colorless solid; Yield: 97% (4 h); m.p. 88–89 °C; ^1^H-NMR (CDCl_3_) δ 7.10 (d, *J* = 7.9 Hz, 2H), 6.98 (d, *J* = 7.9 Hz, 2H), 5.78–5.68 (m, 1H), 5.14–4.93(m, 4H), 4.16 (dd, *J* = 10.8, 3.4 Hz, 1H), 3.81 (s, 3H), 3.74 (s, 3H), 2.56 (dd, *J* = 14.4, 6.5 Hz, 1H), 2.30 (s, 3H) 2.29–2.25 (m, 1H); ^13^C-NMR (CDCl_3_) δ170.4, 170.2, 138.6, 132.1, 131.8, 129.7, 128.8, 120.0, 78.6, 61.1, 53.0 52.9, 46.7, 38.7, 21.2; HRMS (EI) *m*/*z* calc. for C_17_H_21_NO_6_ (M^+^) 335.1369, found 335.1363.

*Dimethyl 2-allyl-2-(1-(4-methoxyphenyl)-2-nitroethyl)malonate* (**3d**): Yellow oil; Yield: 80% (7 h); ^1^H-NMR (CDCl_3_) δ 7.02 (d, *J* = 8.7 Hz, 2H), 6.83 (d, *J* = 8.7 Hz, 2H), 5.76–5.67 (m, 1H), 5.15–4.92 (m, 4H), 4.14 (dd, *J* = 11.0, 3.4 Hz, 1H), 3.81 (s, 3H), 3.77 (s, 3H), 3.74 (s, 3H), 2.57 (dd, *J* =14.4, 8.0 Hz, 1H), 2.29 (dd, *J* =14.4, 8.0 Hz, 1H); ^13^C-NMR (CDCl_3_) δ 170.4, 170.2, 159.9, 132.1, 130.1, 126.7, 120.0, 114.4, 78.6, 61.2, 55.4, 53.0, 52.9, 46.4, 38.7; HRMS (EI) *m*/*z* calc. for C_17_H_21_NO_7_ (M^+^) 351.1318, found 349.1313.

*Dimethyl 2-allyl-2-(2-nitro-1-(thiophen-2-yl)ethyl)malonate* (**3e**): Yellow oil; Yield: 95% (4 h); ^1^H-NMR (CDCl_3_) δ 7.24 (d, *J* = 5.0 Hz, 1H), 6.93–6.90 (m, 1H), 6.89 (d, *J* = 5.0 Hz, 1H), 5.79–5.72 (m, 1H), 5.17–4.92 (m, 4H), 4.52 (dd, *J* = 10.5, 3.1 Hz, 1H), 3.81 (s, 3H), 3.74 (s, 3H), 2.69 (dd, *J* = 14.5, 6.5 Hz, 1H), 2.43 (dd, *J* = 14.5, 8.1 Hz, 1H); ^13^C-NMR (CDCl_3_) δ 169.8, 169.7, 137.1, 131.8, 128.4, 127.0, 126.1, 120.2, 79.6, 61.2, 53.0, 42.7, 38.6; HRMS (EI) *m*/*z* calc. for C_14_H_17_NO_6_S (M^+^) 327.0777, found 327.0771.

*Dimethyl 2-allyl-2-(1-(naphthalen-1-yl)-2-nitroethyl)malonate* (**3f**): Yellow oil; Yield 98% (3.5 h); ^1^H-NMR (CDCl_3_) δ 8.31 (d, *J* = 8.6 Hz, 1H), 7.82 (dd, *J* = 13.7, 8.2 Hz, 1H), 7.59 (td, *J* = 7.4, 0.8 Hz, 1H), 7.50 (t, *J* = 7.5 Hz, 1H), 7.42 (t, *J* = 7.6 Hz, 1H), 7.22 (d, *J* = 7.2 Hz, 1H), 5.75–5.65 (m, 1H), 5.35 (t, *J* = 7.0 Hz, 1H), 5.16 (d, *J* = 7.1 Hz, 2H), 5.00 (d, *J* = 10.1 Hz, 1H), 4.90 (d, *J* = 17.0 Hz, 1H), 3.85 (s, 3H), 3.73 (s, 3H), 2.38 (dd, *J* = 14.2, 6.6 Hz, 1H), 2.23 (dd, *J* = 14.2, 7.9 Hz, 1H); ^13^C-NMR (CDCl_3_) δ 170.6, 170.4, 134.2, 133.1, 132.7, 132.2, 129.4, 129.1, 127.0, 126.3, 125.2, 124.6, 123.5, 119.6, 79.4, 62.8, 52.9, 40.6, 39.1; HRMS (EI) *m*/*z* calc. for C_20_H_21_NO_6_ (M^+^) 371.1369, found 371.1363.

### 3.4. General Procedure for the Preparation of **2a**–**2o** and **4a**–**4f**

A mixture of allyl or propargyl dimethyl malonate (1 mmol) and DBU (1.5 equiv.) in anhydrous dichloromethane (3 mL) was stirred at −78 °C for 20 min under nitrogen. To this mixture, the Yamaguchi reagent (1.5 equiv.) and ZrCl_4_ (10 mol %) were added. The reaction mixture was then stirred at the same temperature for 10 min. then stopped the cooler and continued until the reaction was complete, as indicated by TLC. After the completion of the reaction, the reaction mixture was washed with water, and extracted with CH_2_Cl_2_, washed the organic layer with brine solution. The organic layers were collected, combined, dried over anhydrous magnesium sulfate, and concentrated under reduced pressure. The residue was then purified by column chromatography using EtOAc-hexane to give the desired product.

**2a**, **2j**, **2k**, **4a**–**4f** are known compounds [43].

*Dimethyl-6-(2-bromophenyl)-4H-cyclopenta[c]isoxazole-5*,*5(6H)-dicarboxylate* (**2b**): Brown solid; m.p. 127–129 °C; ^1^H-NMR (CDCl_3_) δ 8.10 (s, 1H), 7.58 (d, *J* = 7.9 Hz, 1H), 7.16 (t, *J* = 7.4 Hz,1H), 7.08 (td, *J* = 7.7, 1.3 Hz, 1H),6.73 (d, *J* = 7.6 Hz, 1H), 5.97 (s, 1H), 3.88 (d, *J* = 17.2 Hz, 1H), 3.80 (s, 3H), 3.29 (s, 3H), 3.25 (d, *J* = 17.2 Hz, 1H); ^13^C-NMR (CDCl_3_) δ 172.8, 170.9, 168.2, 150.8, 137.1, 133.2, 129.8, 129.5, 127.8, 125.5, 121.3, 72.6, 53.7, 52.5, 46.9, 29.9; HRMS (EI) *m*/*z* calc. for C_16_H_14_^79^Br NO_5_ (M^+^) 379.0055, found 379.0050.

### Dimethyl-6-(o-tolyl)-4H-cyclopenta[c]isoxazole-5,5(6H)-dicarboxylate (**2c**): White solid; m.p. 117–118 °C; ^1^H-NMR (CDCl_3_) δ 8.08 (s, 1H), 7.16–7.08 (m, 2H), 7.03 (t, *J* = 7.4 Hz, 1H), 6.97 (d, *J* = 7.4 Hz, 1H), 5.63 (s, 1H), 3.63 (d, *J* = 17.4, 1.5 Hz, 1H), 3.79 (s, 3H), 3.25 (d, *J* = 17.4, 1.4Hz, 1H), 3.19 (s, 3H), 2.49 (s, 3H); ^13^C-NMR (CDCl_3_) δ 173.5, 171.5, 168.5, 150.5, 137.1, 135.8, 130.6, 128.3, 127.9, 126.3, 121.4, 72.9, 53.6, 52.4, 44.0, 29.8, 20.2. HRMS (EI) m/z calc. for C_17_H_17_NO_5_Na (M^+^ + Na) 338.1005, found 338.1015.

*Dimethyl-6-(2-methoxyphenyl)-4H-cyclopenta[c]isoxazole-5*,*5(6H)-dicarboxylate* (**2d**): Pale yellow oil; ^1^H-NMR (CDCl_3_) δ 8.02 (s, 1H), 7.21 (td, *J* = 7.8, 1.5 Hz, 1H), 6.97 (d, *J* = 6.8 Hz, 1H), 6.86–6.81 (m, 2H), 5.63 (s, 1H), 3.88 (dd, *J* = 16.7, 1.2 Hz, 1H), 3.78 (s, 3H), 3.72 (s, 3H), 3.22 (s, 3H), 3.16 (d, *J* = 16.7 Hz, 1H); ^13^C-NMR (CDCl_3_) δ 172.9, 171.6, 168.7, 157.4, 149.8, 130.5, 129.4, 125.8, 121.6, 120.7, 111.1, 72.3, 55.6, 53.5, 52.4, 43.5, 30.4; HRMS (EI) *m*/*z* calc. for C_17_H_17_NO_6_ (M^+^) 331.1056, found 331.1064.

*Dimethyl-6-(4-fluorophenyl)-4H-cyclopenta[c]isoxazole-5*,*5(6H)-dicarboxylate* (**2e**): Pale yellow oil; ^1^H-NMR (CDCl_3_) δ 8.10 (s, 1H), 7.21 (dd, *J* = 8.0, 3.9 Hz, 1H), 6.97 (t, *J* =8.4 Hz, 3H), 5.31 (s, 1H), 3.80 (s, 3H), 3.67 (d, *J* = 16.4 Hz, 1H), 3.24 (s, 3H), 3.11 (d, *J* = 16.8 Hz, 1H); ^13^C-NMR (CDCl_3_) δ 171.8, 171.0, 168.9, 162.6 (d, *J* = 245 Hz), 150.6, 131.8 (d, *J* = 3 Hz), 130.5 (d, *J* = 8 Hz), 121.6, 115.5 (d, *J* = 21 Hz), 72.9, 53.5, 48.0, 29.7; HRMS (EI) *m*/*z* calc. for C_16_H_14_FNO_5_ (M^+^) 319. 0856, found 319.0851.

*Dimethyl-6-(4-chlorophenyl)-4H-cyclopenta[c]isoxazole-5*,*5(6H)-dicarboxylate* (**2f**): Pale yellow solid; m.p. 124–125°C; ^1^H-NMR (CDCl_3_) δ 8.11 (s, 1H), 7.26 (d, *J* = 8.1 Hz, 2H), 7.17 (d, *J* = 8.1 Hz, 2H), 5.30 (s, 1H), 3.81 (s, 3H), 3.68 (d, *J* = 16.6 Hz, 1H), 3.25 (s, 3H), 3.13 (d, *J* = 16.6 Hz, 1H); ^13^C-NMR (CDCl_3_) δ 171.5, 170.9, 168.8, 150.6, 134.6, 134.2, 130.5, 128.7, 121.4, 72.9, 53.6, 52.7, 48.1, 29.7; HRMS (EI) *m/z* calc. for C_16_H_14_^35^ClNO_5_ (M^+^) 335. 056, found 335.0555.

*Dimethyl-6-(4-methoxyphenyl)-4H-cyclopenta[c]isoxazole-5*,*5(6H)-dicarboxylate* (**2g**): White solid; m.p. 111–110 °C; ^1^H-NMR (CDCl_3_) δ 8.06 (s, 1H), 7.09 (d, *J* = 8.6 Hz, 2H), 6.78 (d, *J* = 8.64 Hz, 2H), 5.24 (s, 1H), 3.77 (s, 3H), 3.74 (s, 3H), 3.66 (d, *J* = 16.5 Hz, 1H), 3.23 (s, 3H), 3.10 (d, *J* = 16.5 Hz, 1H); ^13^C-NMR (CDCl_3_) δ 172.2, 171.1, 168.9, 159.4, 150.3, 130.1, 128.0, 121.2, 113.9, 72.8, 55.3, 53.4, 52.5, 48.0, 29.5; HRMS (EI) *m/z* calc. for C_17_H_17_NO_6_ (M^+^) 331.1056, found 331.1050.

*Dimethyl-6-(p-tolyl)-4H-cyclopenta[c]isoxazole-5*,*5(6H)-dicarboxylate* (**2h**): White solid; m.p. 102–103 °C; ^1^H-NMR (CDCl_3_) δ 8.00 (s, 1H), 7.01 (d, *J* = 8.2 Hz, 2H), 6.98 (d, *J* = 8.2 Hz, 2H), 5.19 (s, 1H), 3.72 (s, 3H), 3.63 (d, *J* = 16.6 Hz, 1H), 3.17 (s, 3H), 3.06 (d, *J* = 16.6 Hz, 1H), 2.22 (s, 3H); ^13^C-NMR (CDCl_3_) δ 171.9, 170.9, 168.5, 150.1, 137.6, 132.8, 128.9, 128.6, 121.1, 72.7, 53.2, 52.3, 48.1, 29.4, 21.0; HRMS (EI) *m/z* calc. for C_17_H_17_NO_5_ (M^+^) 315.1107, found 315.1101.

*Dimethyl-6-(4-(methylthio)phenyl)-4H-cyclopenta[c]isoxazole-5*,*5(6H)-dicarboxylate* (**2i**): Orange solid; m.p. 114–116 °C; ^1^H-NMR (CDCl_3_) δ 8.09 (s, 1H), 7.16 (d, *J* = 8.4 Hz, 2H), 7.11 (d, *J* = 8.4 Hz, 2H), 5.27 (s, 1H), 3.79 (s, 3H), 3.69 (dd, *J* = 16.0, 1.0 Hz, 1H), 3.25 (s, 3H), 3.12 (dd, *J* = 16.0, 1.0 Hz, 1H), 2.44 (s, 3H); ^13^C-NMR (CDCl_3_) δ 171.9, 171.1, 168.8, 150.5, 138.7, 132.8, 129.5, 126.6, 121.3, 72.9, 53.5, 52.6, 48.3, 29.7, 15.9; HRMS (EI) *m/z* calc. for C_17_H_17_NO_5_S (M^+^) 347. 0827, found 347.0822.

*Dimethyl-6-(thiophen-2-yl)-4H-cyclopenta[c]isoxazole-5*,*5(6H)-dicarboxylate* (**2j**): White solid; m.p. 129–130 °C; ^1^H-NMR (CDCl_3_) δ 8.08 (s, 1H), 7.23–7.21(m, 1H), 6.94–6.91 (m, 2H), 5.53(s, 1H), 3.81 (s, 3H), 3.68 (dd, *J* = 16.5, 1.2Hz, 1H), 3.41 (s, 3H), 3.14 (dd, *J* = 16.5, 1.2 Hz, 1H); ^13^C-NMR (CDCl_3_) δ 171.5, 170.7, 168.5, 150.6, 137.4, 127.5, 126.8, 125.9, 120.6, 72.9, 53.5, 52.8, 44.1, 29.3; HRMS (EI) *m/z* calc. for C_14_H_13_NO_5_S (M^+^) 307. 0514, found 307.0513.

*Dimethyl-6-(benzo[d]*[1,3]*dioxol-5-yl)-4H-cyclopenta[c]isoxazole-5*,*5(6H)-dicarboxylate* (**2k**): White solid; m.p. 93–94 °C; ^1^H-NMR (CDCl_3_) δ 8.08 (s, 1H), 6.72–6.66 (m, 3H), 5.91–5.90 (m, 2H), 5.23 (s, 1H), 3.79 (s, 3H), 3.66 (d, *J* = 16.5 Hz, 1H), 3.33 (s, 3H), 3.11 (d, *J* = 16.5 Hz, 1H); ^13^C-NMR (CDCl_3_) δ 171.9, 171.0, 168.7, 150.4, 147.7, 129.5, 122.5, 121.2, 109.4, 108.1, 101.2, 72.7, 53.4, 52.6, 48.3, 29.5; HRMS (EI) *m/z* calc. for C_17_H_15_NO_7_ (M^+^) 345. 0849, found 345.0847.

*Dimethyl-6-(naphthalen-1-yl)-4H-cyclopenta[c]isoxazole-5*,*5(6H)-dicarboxylate* (**2l**): White solid; m.p. 131–133 °C; ^1^H-NMR (CDCl_3_) δ 8.39 (d, *J* =5.4 Hz, 1H), 8.15 (s, 1H), 7.83 (d, *J* =8.1 Hz, 1H), 7.75 (d, *J*=8.2 Hz, 1H), 7.58 (t, *J* =6.8 Hz, 1H), 7.49 (t, *J* = 7.6 Hz, 1H), 7.33 (t, *J* = 7.6 Hz, 1H), 6.91 (s, 1H), 6.31 (s, 1H), 3.91 (d, *J* = 16.9 Hz, 1H), 3.83 (s, 3H), 3.31 (d, *J* = 16.9 Hz, 1H), 2.71 (s, 3H); ^13^C-NMR (CDCl_3_) δ 173.3, 171.4, 168.2, 150.6, 133.9, 133.5, 131.9, 128.8, 128.7, 126.6, 126.3, 125.9, 125.3, 124.2, 121.6, 73.0, 53.7, 52.0, 43.2, 29.8; HRMS (EI) *m/z* calc. for C_20_H_17_NO_5_ (M^+^) 351.1106, found 351.1101.

*Dimethyl-6-isobutyl-4H-cyclopenta[c]isoxazole-5*,*5(6H)-dicarboxylate* (**2m**): White solid; m.p. 101–102 °C; ^1^H-NMR (CDCl_3_) δ 7.93 (s, 1H), 3.95 (dd, *J* = 11.7, 4.2 Hz, 1H), 3.76 (s, 3H), 3.75 (s, 3H), 3.46 (dd, *J* = 16.4, 1.5 Hz, 1H), 3.08 (dd, *J* =16.4, 1.5 Hz, 1H), 2.03–1.96 (m, 1H), 1.41 (td, *J* =12.9, 3.7 Hz, 1H), 1.33–1.26 (m, 1H), 1.04 (d, *J* = 6.5 Hz, 3H), 0.93 (d, *J* = 6.5 Hz, 3H); ^13^C-NMR (CDCl_3_) δ 172.4, 171.2, 169.7, 149.5, 120.1, 70.4, 53.3, 52.9, 41.7, 38.0, 29.4, 25.8, 24.0, 21.2; HRMS (EI) *m/z* calc. for C_20_H_17_NO_5_ (M^+^) 281.1263, found 281.1258.

*Dimethyl-6-(2-bromophenyl)-4H-cyclopenta[c]isoxazole-5*,*5(6H)-dicarboxylate* (**2n**): White solid; m.p. 209–211 °C; ^1^H-NMR (CDCl_3_) δ 7.76–7.74 (m, 2H), 7.51–7.41 (m, 3H), 7.31–7.22 (m, 5H), 5.35 (s, 1H), 3.94 (d, *J* = 16.7 Hz, 1H), 3.81 (s, 3H), 3.24 (d, *J* = 16.7 Hz, 1H), 3.22 (s, 3H); ^13^C-NMR (CDCl_3_) δ 173.5, 171.2, 168.8, 161.3, 136.3, 130.1, 129.3, 129.2, 128.2, 127.8, 126.3, 116.9, 73.3, 53.6, 52.6, 49.0, 31.1; HRMS (EI) *m/z* calc. for C_22_H_19_NO_5_ (M^+^) 377.1263, found 377.1265.

## 4. Conclusions

In summary we report on a practical and efficient regioselective synthesis of bicyclic isoxazole/isoxazoline derivatives via intramolecular nitrile oxide cycloaddition reactions using the Yamaguchi reagent and DBU in the presence of ZrCl_4_. The process is straightforward, easy to perform and does not involve the use of costly reagents or catalysts. A wide variety of carbocyclic fused isoxazole and isoxazoline derivatives were synthesized in good to moderate yields.
